# The Combination of Vitamin K_3_ and Vitamin C Has Synergic Activity against Forms of *Trypanosoma cruzi* through a Redox Imbalance Process

**DOI:** 10.1371/journal.pone.0144033

**Published:** 2015-12-07

**Authors:** Vânia Cristina Desoti, Danielle Lazarin-Bidóia, Fabianne Martins Ribeiro, Solange Cardoso Martins, Jean Henrique da Silva Rodrigues, Tania Ueda-Nakamura, Celso Vataru Nakamura, Valdecir Farias Ximenes, Sueli de Oliveira Silva

**Affiliations:** 1 Programa de Pós Graduação em Ciências Farmacêuticas, Universidade Estadual de Maringá, Maringá, PR, Brasil; 2 Programa de Pós Graduação em Ciências Biológicas—Biologia Celular e Molecular, Universidade Estadual de Maringá, Maringá, PR, Brasil; 3 Departamento de Química, Faculdade de Ciências, Universidade Estadual Paulista Julio de Mesquita Filho, Bauru, SP, Brasil; Institut national de la santé et de la recherche médicale—Institut Cochin, FRANCE

## Abstract

Chagas’ disease is an infection that is caused by the protozoan *Trypanosoma cruzi*, affecting millions of people worldwide. Because of severe side effects and variable efficacy, the current treatments for Chagas’ disease are unsatisfactory, making the search for new chemotherapeutic agents essential. Previous studies have reported various biological activities of naphthoquinones, such as the trypanocidal and antitumor activity of vitamin K_3_. The combination of this vitamin with vitamin C exerted better effects against various cancer cells than when used alone. These effects have been attributed to an increase in reactive oxygen species generation. In the present study, we evaluated the activity of vitamin K_3_ and vitamin C, alone and in combination, against *T*. *cruzi*. The vitamin K_3_ + vitamin C combination exerted synergistic effects against three forms of *T*. *cruzi*, leading to morphological, ultrastructural, and functional changes by producing reactive species, decreasing reduced thiol groups, altering the cell cycle, causing lipid peroxidation, and forming autophagic vacuoles. Our hypothesis is that the vitamin K_3_ + vitamin C combination induces oxidative imbalance in *T*. *cruzi*, probably started by a redox cycling process that leads to parasite cell death.

## Introduction

More than one century after the discovery of Chagas’ disease [1], which is caused by the protozoan *Trypanosoma cruzi*, millions of people are still infected worldwide [2]. Although the foci have been reduced, estimates indicate that 50,000–200,000 new cases are diagnosed every year [3]. Chagas’ disease is considered a silent pathology because the first symptoms may appear several years after infection [4]. Only two drugs are available for treatment, benznidazole and nifurtimox, which were developed more than four decades ago, and they have variable efficacy and high toxicity [5]. These drawbacks justify the critical need to identify better treatments for chagasic patients.

Novel compounds, including natural and synthetic drugs and drug combinations, has been intensively studied in an attempt to find the most effective chemotherapies with better activity and fewer side effects [6–8]. The literature presents several studies of compounds with possible efficacy for the treatment of Chagas’ disease. Many of these, such as (-)-elatol, eupomatenoid-5, and naphthoquinones, act by inducing the formation of reactive oxygen and nitrogen species [9–11]. Naphthoquinones are quinoids with a basic skeleton structure that is derived from naphthalene [12]. They possess interesting biological and pharmacological activity [13–15]. For example, 2-methyl-1,4-naphthoquinone (menadione or vitamin K_3_ [VK_3_]) has anticancer effects [16, 17]. The combination of VK_3_ and ascorbic acid (vitamin C [VC]) has been shown to have tumor-specific antitumor effects against many cancer cells both *in vitro* and *in vivo* [18–25]. The mechanism of cell death that is induced by this combination of vitamins is associated with oxidative imbalance that is generated through a redox cycling process [26, 27], with apparent activity at concentrations that are 10- to 50-times lower those of the individual vitamins alone [28, 29].

Considering the redox imbalance that is induced by VC + VK_3_ [26, 27] and the different antioxidant capacity of *T*. *cruzi* parasites compared with mammals [30, 31], we investigated the activity of this vitamin combination against the epimastigote, trypomastigote, and amastigote forms of this protozoan. The results showed that the VC + VK_3_ combination has synergistic effects against all three forms of *T*. *cruzi*. Additionally, based on the morphological and ultrastructural alterations that were observed and the results of different probes and compounds that were used to evaluate the effects of VC + VK_3_ on *T*. *cruzi*, we hypothesized that this vitamin combination may trigger an initial processes that are related to increases in the generation of reactive species and reduction of reduced thiol levels, followed by irreversible oxidative imbalance that triggers alterations that are incompatible with *T*. *cruzi* survival.

## Materials and Methods

### Chemicals

VC, VK_3_, 2’,7’-dichlorodihydrofluorescein diacetate (H_2_DCFDA), 5,5’-dithiobis-(2-nitrobenzoic acid) (DTNB), monodansylcadaverine (MDC), and wortmannin (WTM) were purchased from Sigma-Aldrich (St. Louis, MO, USA). Dulbecco’s modified Eagle’s medium (DMEM), fetal bovine serum (FBS), and Giemsa were obtained from Invitrogen (Grand Island, NY, USA). Propidium iodide/RNase A (PI-RNase A), diphenyl-1-pyrenylphosphine (DPPP), and 4-amino-5-methylamino-2’,7’-difluorofluorescein (DAF-FM) diacetate were obtained from Invitrogen (Eugene, OR, USA). All of the other reagents were of analytical grade.

### Parasites and cell culture

All of the experiments were performed using the Y strain of *T*. *cruzi* [32]. Epimastigote forms were axenically maintained at 28°C with weekly transfers in liver infusion tryptose (LIT) medium supplemented with 10% heat-inactivated FBS, pH 7.4 [33]. Trypomastigote and amastigote forms were obtained from the previously infected monolayers of LLCMK_2_ cells (epithelial cells of monkey kidney [*Macaca mulatta*]; CCL-7; American Type Culture Collection, Rockville, MD, USA) in DMEM supplemented with 2 mM L-glutamine and 10% FBS, buffered with sodium bicarbonate in a 5% CO_2_ air mixture at 37°C.

### *In vitro* assay of vitamin combination

To evaluate the effects of the VK_3_ + VC combination on epimastigotes, trypomastigotes, and amastigotes, we applied the Combination Index method as described by Chou and Talalay [34] and reviewed by Zhao *et al*. [35]. The experimental design consisted of combinations of at least four concentrations of each vitamin arranged on a checkerboard at a 1:2 concentration ratio.

Epimastigote forms (1 × 10^6^ parasites/ml) in the exponential growth phase were resuspended in LIT medium supplemented with 10% FBS. The vitamins were added to the cell suspension, alone or in combination (1.0–9.0 μM VK_3_ and 0.35–2.84 mM VC), in 24-well plates and incubated at 28°C. The number of epimastigote forms was determined by counting in a Neubauer hemocytometer after 96 h.

To evaluate activity against trypomastigote forms, parasites were obtained from the supernatant of infected LLCMK_2_ cells. Trypomastigote forms (1 × 10^7^ parasites/ml) were resuspended in the presence of DMEM supplemented with 10% FBS and different concentrations of both vitamins, alone or in combination (0.14–4.65 μM VK_3_ and 0.09–1.42 mM VC), in 96-well plates. Parasites were incubated for 24 h at 37°C in a 5% CO_2_ atmosphere. After incubation, the viability of the parasites was determined by examining mobility under a light microscope (Olympus CX31) using the Pizzi-Brener method [36].

To evaluate activity against intracellular amastigote forms, LLCMK_2_ cells (2.5 × 10^5^ cells/ml) were harvested, resuspended in DMEM supplemented with 10% FBS, and plated in 24-well plates that contained round glass coverslips. When confluent growth was achieved, the cells were infected with trypomastigotes (1 × 10^7^ parasites/ml) that were obtained from preinfected cultures. After 24 h, the medium that contained the parasites was removed. The cells were then washed in phosphate-buffered saline (PBS), and DMEM with different concentrations of both vitamins, alone or in combination (0.29–4.65 μM VK_3_ and 0.18–2.84 mM VC), was added. The cells were maintained for 96 h at 37°C in a 5% CO_2_ atmosphere. Afterward, the glass coverslips were subjected to fixation with methanol and Giemsa staining and permanently prepared with Entellan (Merck, Darmstadt, Germany). The number of infected cells and amastigotes was determined by randomly counting 200 cells. The results were calculated as the survival index, which was obtained by multiplying the percentage of infected cells by the number of amastigotes per infected LLCMK_2_ cell and then determining the percentage of inhibition. The treated groups were compared with the untreated control, the survival index observed in the control without treatment was considered 100%.

The data were calculated and mathematically expressed as the Combination Index: *CI = (IC_50_ VK_3_ combined / IC_50_ VK_3_ alone) + (IC_50_ VC combined / IC_50_ VC alone)* for epimastigotes and amastigotes and *CI = (EC_50_ VK_3_ combined / EC_50_ VK_3_ alone) + (EC_50_ VC combined / EC_50_ VC alone)* for trypomastigotes. The numerators are the concentrations of each vitamin that in combination are active against 50% of the parasites, and the denominators are the concentrations that have the same effect for each vitamin alone. The IC_50_ is the inhibitory concentration, and the EC_50_ is the effective concentration. The interpretation of the CI was based on the broadly used specifications that were established by Chou and Talalay [34]. When CI = 1, the combination is additive. When CI < 1, the combination is synergistic. When CI > 1, the combination is antagonistic. The data were also graphically expressed as isobolograms by plotting concentrations of vitamins that alone or in combination induced activity against 50% of the forms of the parasite.

### Scanning electron microscopy analysis

For scanning electron microscopy (SEM), epimastigote forms (1 × 10^6^ parasites/ml) were treated with 1.90 μM VK_3_ and 0.61 mM VC, alone or in combination, for 72 h at 28°C. Trypomastigote forms (1 × 10^7^ parasites/ml) were treated with 0.35 μM VK_3_ and 0.20 mM VC, alone or in combination, for 24 h at 37°C in a 5% CO_2_ atmosphere. Intracellular amastigotes were treated with 0.30 μM VK_3_ and 0.18 mM VC, alone or in combination, for 24 h at 37°C in a 5% CO_2_ atmosphere. After incubation, the parasites were harvested, washed twice in PBS, and fixed with 2.5% glutaraldehyde in 0.1 M sodium cacodylate buffer at 4°C. The parasites were then placed on a glass support that was covered with poly-L-lysine, dehydrated in an ascending series of ethanol, critical-point dried with CO_2_, coated with gold, and observed in a Shimadzu SS-550 scanning electron microscope. For intracellular amastigotes, we used the fracture tape method.

### Transmission electron microscopy analysis

For transmission electron microscopy (TEM), epimastigote forms (1 × 10^6^ parasites/ml) were treated with 1.90 μM VK_3_ and 0.61 mM VC, alone or in combination, for 72 h at 28°C. Trypomastigote forms (1 × 10^7^ parasites/ml) were treated with 0.35 μM VK_3_ and 0.20 mM VC, alone or in combination, for 24 h at 37°C in a 5% CO_2_ atmosphere. Intracellular amastigotes were treated with 0.30 μM VK_3_ and 0.18 mM VC, alone or in combination, for 24 h at 37°C in a 5% CO_2_ atmosphere. After incubation, the parasites were harvested, washed twice in PBS, fixed with 2.5% glutaraldehyde in 0.1 M sodium cacodylate buffer at 4°C, and postfixed in a solution of 1% OsO_4_, 0.8% potassium ferrocyanide, and 10.0 mM CaCl_2_ in 0.10 M cacodylate buffer. The samples were then dehydrated in an increasing acetone gradient and embedded in Polybed 812 resin. Ultrathin sections were then obtained, stained with uranyl acetate and lead citrate, and observed in a JEOL JM 1400 transmission electron microscope.

### Detection of total reactive oxygen species

The production of total reactive oxygen species (total ROS) was evaluated in parasitic forms after exposure to VK_3_ and VC using the probe H_2_DCFDA. Epimastigote forms (1 × 10^6^ parasites/ml) were evaluated after exposure to 1.90 μM VK_3_ and 0.61 mM VC, alone and in combination, for 24 h at 28°C. Trypomastigote forms (1 × 10^7^ parasites/ml) were evaluated after exposure to 0.35 μM VK_3_ and 0.20 mM VC, alone and in combination, for 24 h at 37°C in a 5% CO_2_ atmosphere. Amastigote forms (1 × 10^7^ parasites/ml) were evaluated after exposure to 0.30 μM VK_3_ and 0.18 mM VC, alone and in combination, for 24 h at 37°C in a 5% CO_2_ atmosphere. Hydrogen peroxide (H_2_O_2_; 20.0 μM) was used as a positive control. Afterward, the parasites were centrifuged, washed, and resuspended in PBS. Parasites were loaded with 10.0 μM of the permeant probe H_2_DCFDA in the dark for 45 min. Total ROS were measured as an increase in fluorescence that is caused by the conversion of nonfluorescent dye to highly fluorescent 2’,7’-dichlorofluorescein (DCF) in a fluorescence microplate reader (Victor X3, PerkinElmer) at λ_excitation_ = 488 nm and λ_emission_ = 530 nm.

### Detection of nitric oxide

The production of nitric oxide (NO) was evaluated in parasitic forms after exposure to VK_3_ and VC using the probe DAF-FM diacetate. Epimastigote forms (1 × 10^6^ parasites/ml) were evaluated after exposure to 1.90 μM VK_3_ and 0.61 mM VC, alone and in combination, for 24 h at 28°C. Trypomastigote forms (1 × 10^7^ parasites/ml) were evaluated after exposure to 0.35 μM VK_3_ and 0.20 mM VC, alone and in combination, for 24 h at 37°C in a 5% CO_2_ atmosphere. Amastigote forms (1 × 10^7^ parasites/ml) were evaluated after exposure to 0.30 μM VK_3_ and 0.18 mM VC, alone and in combination, for 24 h at 37°C in a 5% CO_2_ atmosphere. Afterward, the parasites were centrifuged, washed, and resuspended in PBS. The parasites were then loaded with 1.0 μM of the probe DAF-FM diacetate in the dark for 30 min at 37°C. Afterward, the parasites were washed and resuspended in PBS and incubated for an additional 15 min. DAF-FM diacetate is cell-permeant that is deacetylated inside cells to become DAF-FM. This compound in the presence of NO is converted to form fluorescent benzotriazole, which was detected in a fluorescence microplate reader (Victor X3, PerkinElmer) at λ_excitation_ = 495 nm and λ_emission_ = 515 nm.

### Determination of reduced thiol levels

Trypanothione reductase (TR) activity plays an important role in the antioxidant activity of trypanosomatids. Its depletion decreases reduced thiol level [37]. Reduced thiol levels were evaluated in parasitic forms after exposure to VK_3_ and VC. Epimastigote forms (1 × 10^6^ parasites/ml) were evaluated after exposure to 1.90 μM VK_3_ and 0.61 mM VC, alone and in combination, for 24 h at 28°C. Trypomastigote forms (1 × 10^7^ parasites/ml) were evaluated after exposure to 0.35 μM VK_3_ and 0.20 mM VC, alone and in combination, for 24 h at 37°C in a 5% CO_2_ atmosphere. Amastigote forms (1 × 10^7^ parasites/ml) were evaluated after exposure to 0.30 μM VK_3_ and 0.18 mM VC, alone and in combination, for 24 h at 37°C in a 5% CO_2_ atmosphere. Afterward, the parasites were centrifuged. Tris-HCl buffer (10 mM, pH 2.5) was then added and the cells were sonicated. Acidic pH was used during sonication to prevent oxidation of the free thiol groups. Cellular debris was removed by centrifugation, and 100 μl of the supernatant and 100 μl of 500.0 mM phosphate buffer (pH 7.5) were taken from each well, followed by the addition of 20 μl of 1.0 mM DTNB. Free thiol levels were determined using DTNB. Absorbance was measured at 412 nm [37].

### Lipid peroxidation assays

Lipid peroxidation was evaluated in parasitic forms after exposure to VK_3_ and VC. The extent of lipid peroxidation was evaluated by DPPP, which is essentially nonfluorescent until it is oxidized to a phosphine oxide (DPPP-oxide) by peroxides. Epimastigote forms (1 × 10^6^ parasites/ml) were evaluated after exposure to 1.90 μM VK_3_ and 0.61 mM VC, alone and in combination, for 24 h at 28°C. Trypomastigote forms (1 × 10^7^ parasites/ml) were evaluated after exposure to 0.35 μM VK_3_ and 0.20 mM VC, alone and in combination, for 24 h at 37°C in a 5% CO_2_ atmosphere. Amastigote forms (1 × 10^7^ parasites/ml) were evaluated after exposure to 0.30 μM VK_3_ and 0.18 mM VC, alone and in combination, for 24 h at 37°C in a 5% CO_2_ atmosphere. After incubation, the parasites were centrifuged, washed, and resuspended in PBS. The parasites were loaded with 50.0 μM DPPP in the dark for 15 min. The direct fluorometric detection was measured as an increase in the fluorescence of the DPPP oxide in a fluorescence microplate reader (Victor X3, PerkinElmer) at λ_excitation_ = 355 nm and λ_emission_ = 460 nm.

We also determined the amount of thiobarbituric acid-reactive substances (TBARS) in terms of malondialdehyde (MDA) levels. Epimastigote forms (14 mg/ml) were evaluated after exposure to 1.90 μM VK_3_ and 0.61 mM VC, alone and in combination, for 24 h at 28°C. Trypomastigote forms (14 mg/ml) were evaluated after exposure to 0.35 μM VK_3_ and 0.20 mM VC, alone and in combination, for 24 h at 37°C in a 5% CO_2_ atmosphere. Amastigote forms (14 mg/ml) were evaluated after exposure to 0.30 μM VK_3_ and 0.18 mM VC, alone and in combination, for 24 h at 37°C in a 5% CO_2_ atmosphere. After incubation, the samples (0.50 mg protein) were heated in a solution that contained 0.37% thiobarbituric acid, 15.0% trichloroacetic acid, and 0.25 N HCl for 45 min at 90–95°C. After cooling, absorbance was read at 532 nm, and the TBARS concentration was calculated based on an ε value of 153,000 M^-1^cm^-1^ [38].

### Evaluation of cell cycle

The cell cycle was evaluated in epimastigote forms of *T*. *cruzi* after exposure to VK_3_ and VC. Epimastigote forms (1 × 10^6^ parasites/ml) were evaluated after exposure to 1.90 μM VK_3_ and 0.61 mM VC, alone and in combination, for 24 h at 28°C. After incubation, the cells were fixed in 70% cold methanol at 4°C for 1 h. Afterward, the parasites were washed in PBS, and 10 μl of PI-RNase A was added, followed by incubation at 37°C for 45 min. Data acquisition and analysis were performed using a FACSCalibur flow cytometer equipped with CellQuest software. A total of 10,000 events were acquired in the region that corresponded to the parasites. The percentages of cells in each stage of the cell cycle were determined.

### Evaluation of autophagic vacuoles

Autophagic vacuoles were evaluated in parasitic forms after exposure to VK_3_ and VC using MDC labeling, a fluorescent probe that accumulates in autophagic vacuoles [39]. Epimastigote forms (1 × 10^6^ parasites/ml) were evaluated after exposure to 1.90 μM VK_3_ and 0.61 mM VC, alone and in combination, for 24 h at 28°C. Trypomastigote forms (1 × 10^7^ parasites/ml) were evaluated after exposure to 0.35 μM VK_3_ and 0.20 mM VC, alone and in combination, for 24 h at 37°C in a 5% CO_2_ atmosphere. Amastigote forms (1 × 10^7^ parasites/ml) were evaluated after exposure to 0.30 μM VK_3_ and 0.18 mM VC, alone and in combination, for 24 h at 37°C in a 5% CO_2_ atmosphere. The cells were then incubated with 0.05 mM MDC in PBS for 1 h at 37°C. After incubation, the cells were washed twice in PBS. MDC staining was analyzed using an Olympus BX51 fluorescence microscope, images were captured using a UC30 camera, and fluorescence intensity was evaluated by ImageJ 1.44o. In some of the experiments, the parasites were pretreated with 500.0 nM WTM before the induction of autophagy [40]. This compound is a potent phosphatidylinositol 3-kinase inhibitor, an enzyme that is involved in the regulation of autophagy [41].

### Statistical analyses

All of the quantitative experiments were performed at least three times on independent occasions. Data were evaluated using one- or two-way analysis of variance (ANOVA) with significant differences among means identified by Tukey and Bonferroni *post hoc* tests, respectively. Values of *p* ≤ 0.05 were considered statistically significant. The statistical analyses were performed using GraphPad software.

## Results

### Vitamin K_3_ + vitamin C combination induces trypanocidal effect

**Fig 1:**
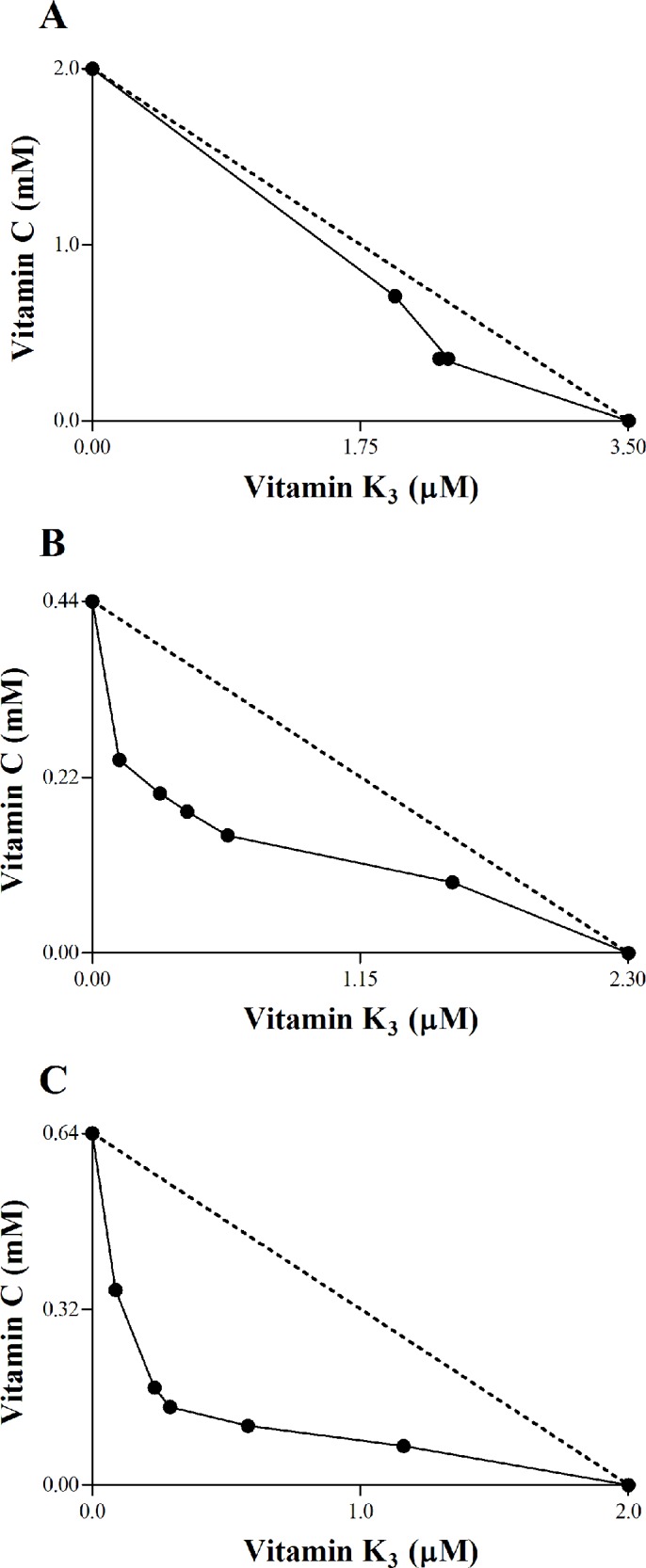
Combination effect of vitamin K_3_ and vitamin C. The isobolograms illustrate the effect of the VK_3_ + VC combination against epimastigote forms (A), trypomastigote forms (B), and intracellular amastigote forms (C). The dotted lines correspond to an additive effect. Points below the line indicate a synergistic effect. Points above the line indicate an antagonistic effect. The points show median values.

We initially investigated the effect of VK_3_ and VC, alone and in combination, on the growth of epimastigotes and intracellular amastigotes and viability of trypomastigotes using *in vitro* assays. We found that this vitamin combination had dose-dependent and robust synergistic effects on the three forms of *T*. *cruzi* (Fig 1). A CI of 0.85 against epimastigote forms was found, with a concave curve profile on the isobologram (Fig 1A), confirming a synergistic interaction. A CI of 0.61 against trypomastigote forms was found, with the same curve shape on the isobologram, also confirming a synergistic interaction (Fig 1B). Furthermore, VK_3_ + VC reduced the percentage of infected LLCMK_2_ cells and the mean number of intracellular amastigotes per infected LLCMK_2_ cell. These data were reflected by a concave curve on the isobologram, with a CI of 0.43 (Fig 1C). The combinations that presented synergistic effects on 50% of the parasites were the following: 1.90 μM VK_3_ + 0.61 mM VC, 0.35 μM VK_3_ + 0.20 mM VC, and 0.30 μM VK_3_ + 0.18 mM VC for the epimastigote, trypomastigote, and amastigote forms, respectively (see S1 Table).

### Vitamin K_3_ + vitamin C combination induces alterations in *T*. *cruzi* morphology and ultrastructure

**Fig 2:**
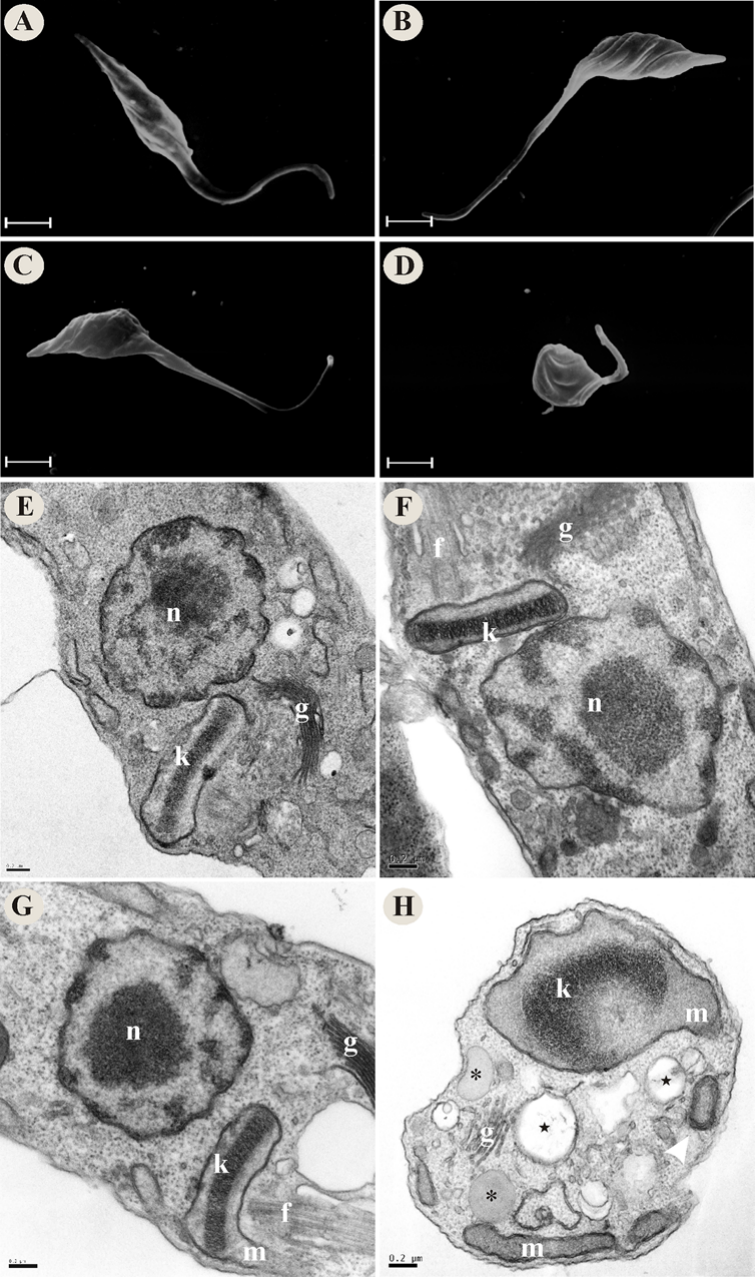
Morphological and ultrastructural alterations in epimastigote forms of *Trypanosoma cruzi* that were treated with vitamin K_3_ and vitamin C alone and combined at 72 h. SEM images: (A) Untreated epimastigote forms. (B) Epimastigote forms that were treated with 1.90 μM VK_3_. (C) Epimastigote forms that were treated with 0.61 mM VC. (D) Epimastigote forms that were treated with 1.90 μM VK_3_ + 0.61 mM VC. TEM images: (E) Untreated epimastigote forms. (F) Epimastigote forms that were treated with 1.90 μM VK_3_. (G) Epimastigote forms that were treated with 0.61 mM VC. (H) Epimastigote forms that were treated with 1.90 μM VK_3_ + 0.61 mM VC. Star, cytoplasmic vacuoles; asterisk, lipid bodies; white arrowhead, myelin-like structure; f, flagellum; g, Golgi complex; k, kinetoplast; m, mitochondrion; n, nucleus. Scale bars = 2 μm in A-D and 0.2 μm in E-H.

**Fig 3:**
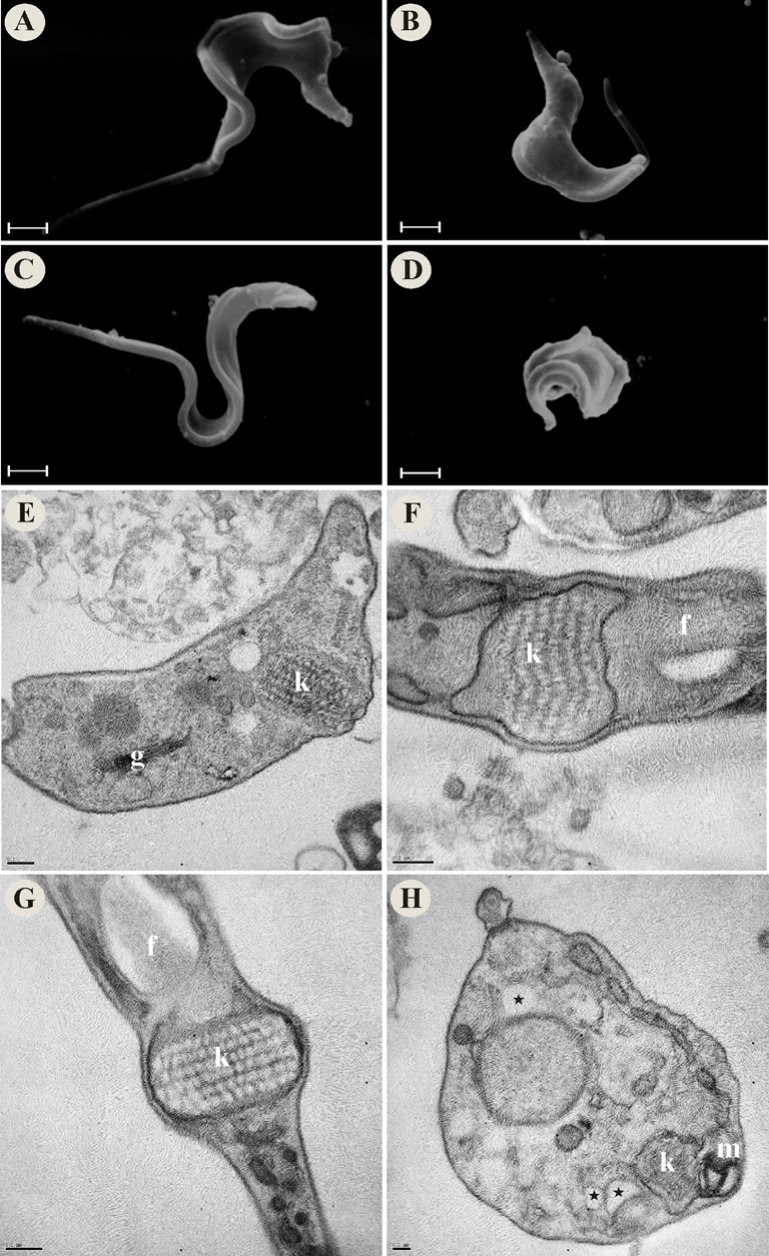
Morphological and ultrastructural alterations in trypomastigote forms of *Trypanosoma cruzi* that were treated with vitamin K_3_ and vitamin C alone and combined at 24 h. SEM images: (A) Untreated trypomastigote forms. (B) Trypomastigote forms that were treated with 0.35 μM VK_3_. (C) Trypomastigote forms that were treated with 0.20 mM VC. (D) Trypomastigote forms that were treated with 0.35 μM VK_3_ + 0.20 mM VC. TEM images: (E) Untreated trypomastigote forms. (F) Trypomastigote forms that were treated with 0.35 μM VK_3_. (G) Trypomastigote forms that were treated with 0.20 mM VC. (H) Trypomastigote forms that were treated with 0.35 μM VK_3_ + 0.20 mM VC. Star, cytoplasmic vacuoles; f, flagellum; g, Golgi complex; k, kinetoplast; m, mitochondrion. Scale bars = 1 μm in A-D and 0.2 μm in E-H.

**Fig 4:**
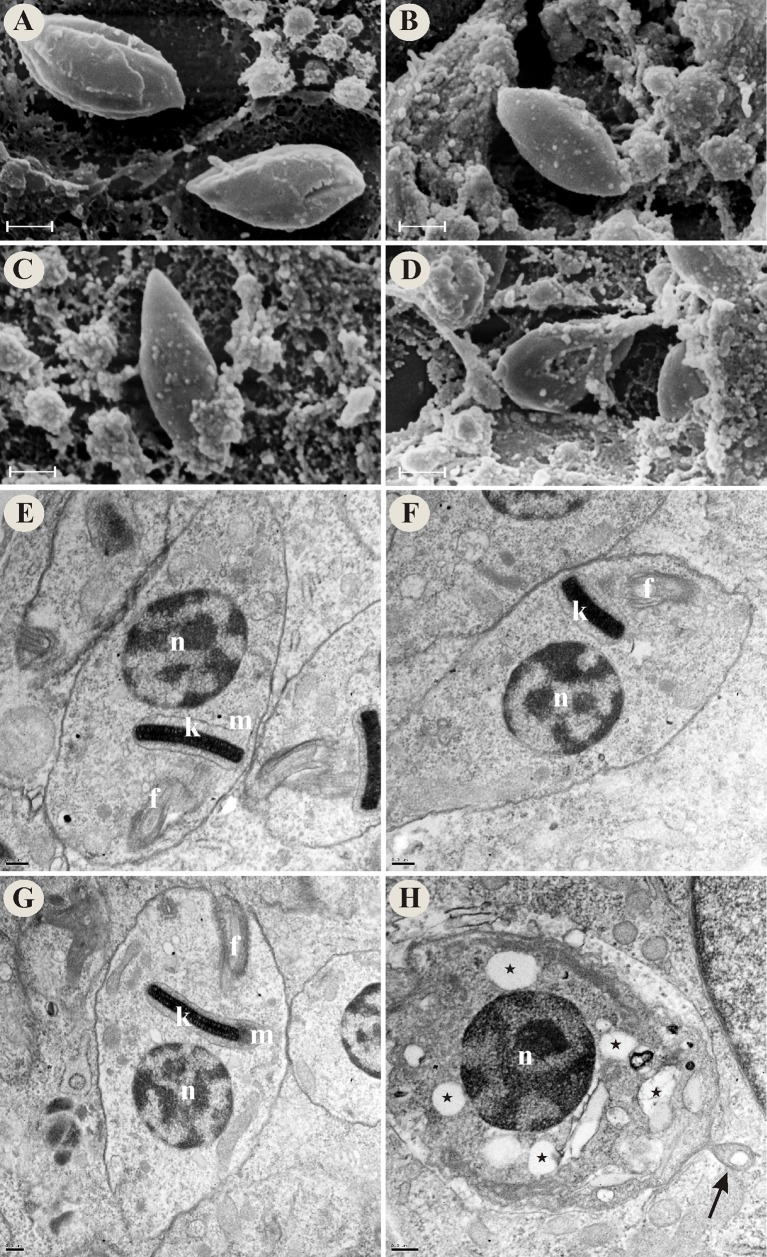
Morphological and ultrastructural alterations in intracellular amastigote forms of *Trypanosoma cruzi* that were treated with vitamin K_3_ and vitamin C alone and combined at 24 h. SEM images: (A) Untreated intracellular amastigote forms. (B) Intracellular amastigote forms that were treated with 0.30 μM VK_3_. (C) Intracellular amastigote forms that were treated with 0.18 mM VC. (D) Intracellular amastigote forms that were treated with 0.30 μM VK_3_ + 0.18 mM VC. TEM images: (E) Untreated intracellular amastigote forms. (F) Intracellular amastigote forms that were treated with 0.30 μM VK_3_. (G) Intracellular amastigote forms that were treated with 0.18 mM VC. (H) Intracellular amastigote forms that were treated with 0.30 μM VK_3_ + 0.18 mM VC. Star, cytoplasmic vacuoles; arrow, blebs in plasma membrane; f, flagellum; k, kinetoplast; m, mitochondrion; n, nucleus. Scale bars = 1 μm in A-D and 0.2 μm in E-H.

We evaluated the morphological and ultrastructural effects of VK_3_ and VC, alone and in combination, on *T*. *cruzi* using SEM and TEM. By SEM was observed that the parasites incubated with combinations of VK_3_ + VC exhibited alterations in the shape of the parasites, including rounding (Figs 2D and 3D) and change of the plasma membrane (Fig 4D). In amastigotes a rounding of the body also was observed (data not shown). In contrast, the parasites that were treated with the same concentrations of the vitamins alone (Figs 2B and 2C, 3B and 3C, 4B and 4C) had a typical shape that was similar to untreated parasites (Figs 2A, 3A and 4A).

By TEM was observed that untreated parasites and parasites that were treated with the vitamins alone generally exhibited a normal organelle ultrastructure, such as prominent nucleus and mitochondrion, and cellular membranes with preserved structures (Figs 2E–2G, 3E–3G, and 4E–4G). The parasites that were treated with the VK_3_ + VC combination exhibited swelling in the mitochondrion-kinetoplast region (Fig 2H), myelin-like structure (Fig 2H), cytoplasmic vacuoles (Figs 2H, 3H and 4H), the formation of intracellular lipid bodies (Fig 2H), the formation of blebs in the parasite membrane (Fig 4H), separation between the membrane and cytoplasm, and membranes within the mitochondrion (data not shown).

### Vitamin K_3_ + vitamin C combination increases the production of total reactive oxygen species and nitric oxide

**Fig 5:**
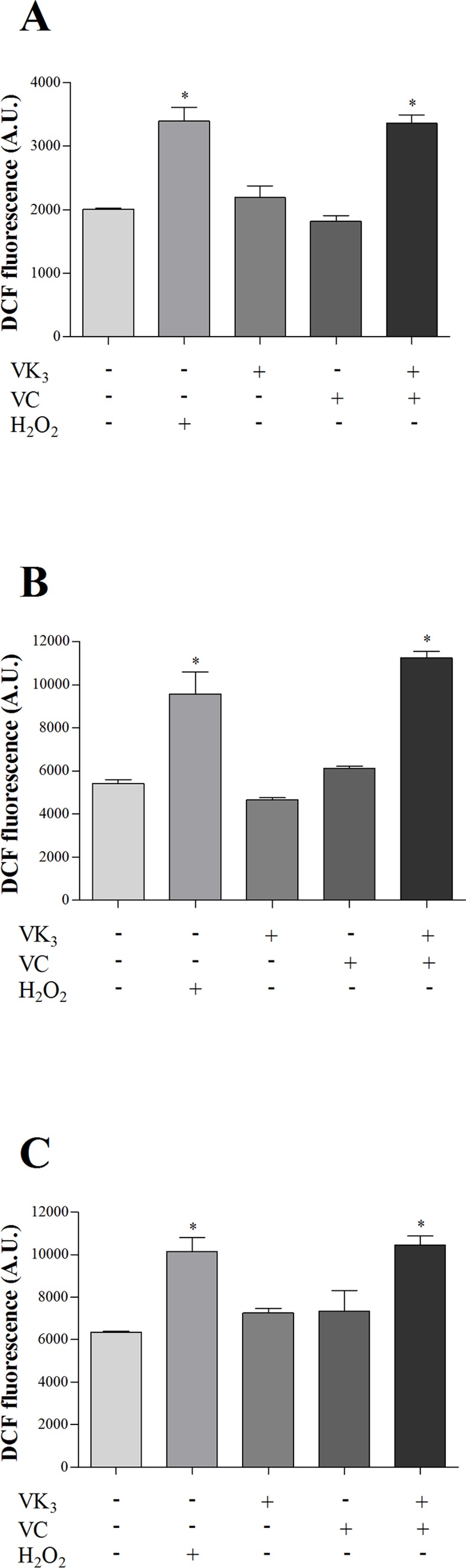
Total ROS production in parasitic forms of *Trypanosoma cruzi* that were treated with vitamin K_3_ and vitamin C, alone and combined, for 24 h using H_2_DCFDA labeling. (A) Epimastigote forms that were treated with 1.90 μM VK_3_ and 0.61 mM VC, alone and combined. (B) Trypomastigote forms that were treated with 0.35 μM VK_3_ and 0.20 mM VC, alone and combined. (C) Amastigote forms that were treated with 0.30 μM VK_3_ and 0.18 mM VC, alone and combined. H_2_O_2_ used as a positive control is also shown. Total ROS were measured as an increase in fluorescence that is caused by the conversion of nonfluorescent dye to fluorescent DCF. The results are expressed as the mean fluorescence (in arbitrary units [A.U.] ± SE) of at least three independent experiments. * Indicate significant differences compared with the control group (untreated cells; *p* ≤ 0.05).

**Fig 6:**
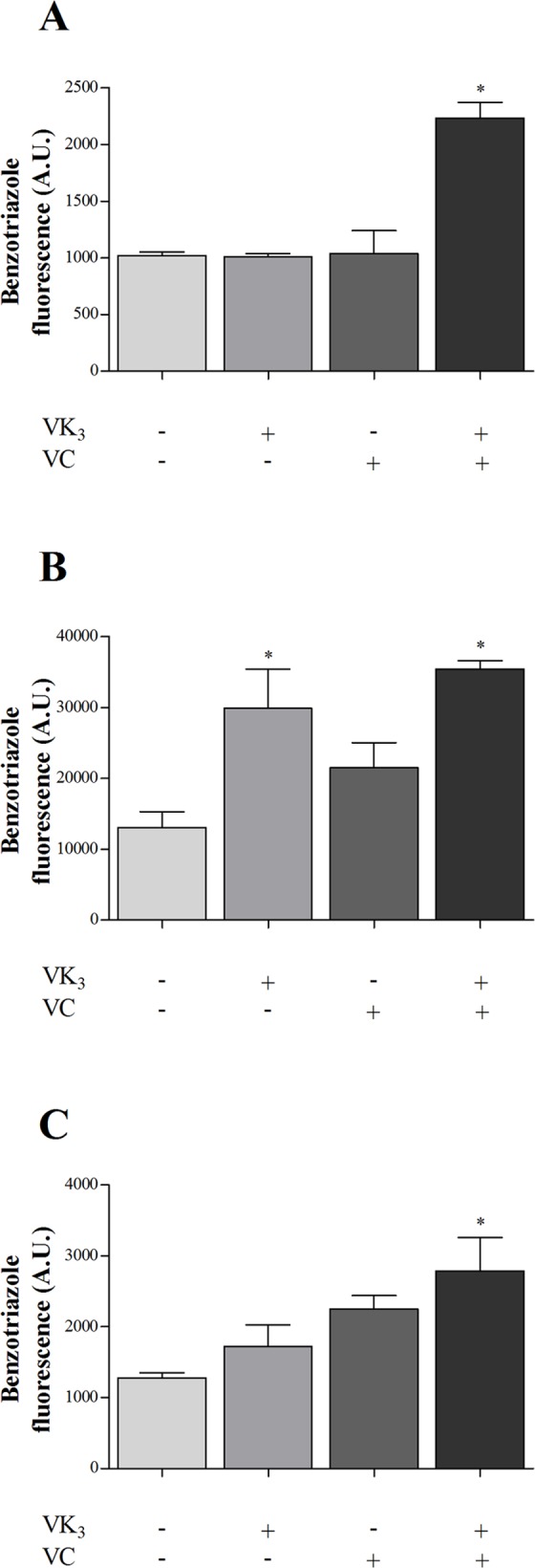
Nitric oxide production in parasitic forms of *Trypanosoma cruzi* that were treated with vitamin K_3_ and vitamin C, alone and combined, for 24 h using DAF-FM diacetate labeling. (A) Epimastigote forms that were treated with 1.90 μM VK_3_ and 0.61 mM VC, alone and combined. (B) Trypomastigote forms that were treated with 0.35 μM VK_3_ and 0.20 mM VC, alone and combined. (C) Amastigote forms that were treated with 0.30 μM VK_3_ and 0.18 mM VC, alone and combined. The NO was measured as an increase in fluorescence that is caused by the conversion of DAF-FM to form fluorescent benzotriazole. The results are expressed as the mean fluorescence (in arbitrary units [A.U.] ± SE) of at least three independent experiments. * Indicate significant differences compared with the control group (untreated cells; *p* ≤ 0.05).

We also investigated the effects of VK_3_ and VC, alone and in combination, on the generation of total ROS (Fig 5) and NO (Fig 6) using H_2_DCFDA and DAF-FM diacetate, respectively. The parasites that were treated with the VK_3_ + VC combination exhibited a higher DCF fluorescence signal compared with treatment with either vitamin alone and untreated parasites (Fig 5). This signal was observed in all three forms of the parasite, and the concentrations of the vitamin combination that exerted effects in 50% of the parasites (1.90 μM VK_3_ + 0.61 mM VC for epimastigotes, 0.35 μM VK_3_ + 0.20 mM VC for trypomastigotes, and 0.30 μM VK_3_ + 0.18 mM VC for amastigotes) caused increases in total ROS production of 67.0%, 108.0%, and 65.0%, respectively, compared with the control group (Fig 5A–5C). The positive control (H_2_O_2_) also increased ROS production in epimastigotes (69.0% Fig 5A), trypomastigotes (77.0% Fig 5B), and amastigotes (60.4% Fig 5C).

The VK_3_ + VC combination increased NO production by more than 100.0% in epimastigotes, trypomastigotes, and amastigotes compared with the control group (Fig 6A–6C). The VK_3_ alone also induced increased in NO production (Fig 6B).

### Vitamin K_3_ + vitamin C combination decreases reduced thiol levels

**Fig 7:**
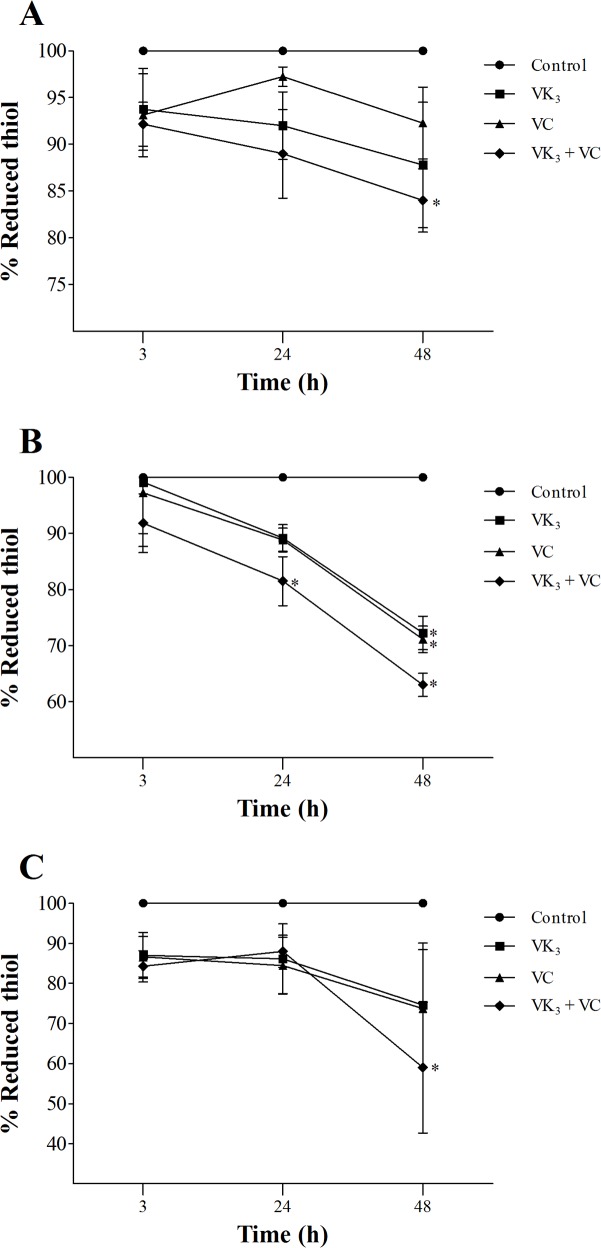
Reduced thiol levels in parasitic forms of *Trypanosoma cruzi* that were treated with vitamin K_3_ and C, alone and combined, for 3, 24, and 48 h using DTNB. (A) Epimastigote forms that were treated with 1.90 μM VK_3_ and 0.61 mM VC, alone and combined. (B) Trypomastigote forms that were treated with 0.35 μM VK_3_ and 0.20 mM VC, alone and combined. (C) Amastigote forms that were treated with 0.30 μM VK_3_ and 0.18 mM VC, alone and combined. The results are expressed as the mean percentage (± SE) of at least three independent experiments. * Indicate significant differences compared with the control group (untreated cells; *p* ≤ 0.05).

We tested whether VK_3_ and VC, alone and in combination, decrease reduced thiol levels in *T*. *cruzi* using DTNB. A significant decrease in total reduced thiol levels was observed in the three forms of *T*. *cruzi* that were treated with vitamin combination compared with the control group at 48 h of treatment (Fig 7). The treatment caused 16.0%, 37.0%, and 41.0% decreases in total reduced thiol levels in epimastigotes, trypomastigotes, and amastigotes, respectively. In trypomastigotes, a significant decrease in total reduced thiol levels (18.5%) was also observed with vitamin combination compared with the control group, even at 24 h of treatment (Fig 7B).

### Vitamin K_3_ + vitamin C combination increases lipid peroxidation

**Fig 8:**
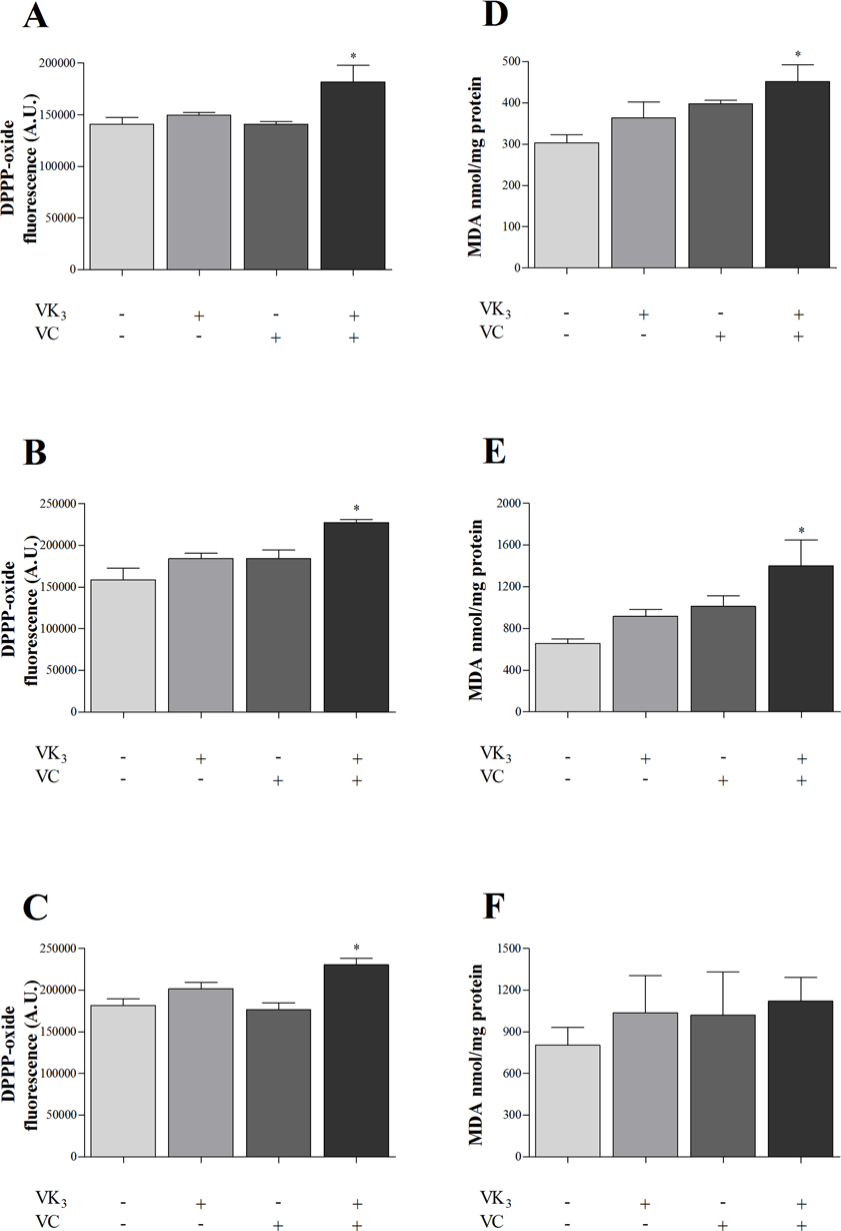
Lipid peroxidation in parasitic forms of *Trypanosoma cruzi* that were treated with vitamin K_3_ and vitamin C, alone and combined, for 24 h. (A-C) Lipid peroxidation, determined by DPPP labeling. The results are expressed as the mean fluorescence (in arbitrary units [A.U.] ± SE) of at least three independent experiments. (D-F) Lipid peroxidation, determined as the amount of TBARS in terms of MDA levels. The results are expressed as the mean MDA nmol/mg protein (± SE) of at least three independent experiments. (A, D) Epimastigote forms that were treated with 1.90 μM VK_3_ and 0.61 mM VC, alone and combined. (B, E) Trypomastigote forms that were treated with 0.35 μM VK_3_ and 0.20 mM VC, alone and combined. (C, F) Amastigote forms that were treated with 0.30 μM VK_3_ and 0.18 mM VC, alone and combined. * Indicate significant differences compared with the control group (untreated cells; *p* ≤ 0.05).

We quantified lipid peroxidation in the three forms of *T*. *cruzi* that were treated with VK_3_ and VC, alone and in combination, using DPPP-labeled cells (Fig 8A–8C) and TBARS (in terms of MDA levels; Fig 8D–8F). Both protocols revealed an increase in lipid peroxidation in parasites that were treated with the vitamin combination compared with the control group. These increases were > 27.0% (Fig 8A–8C) and > 39.0% (Fig 8D–8F) in the three forms of the parasite compared with the control group. However, although not significant, we observed an increase in TBARS in amastigotes that were treated with the vitamin combination (Fig 8F).

### Vitamin K_3_ + vitamin C combination induces sub-G0/G1-phase cell-division arrest

**Fig 9:**
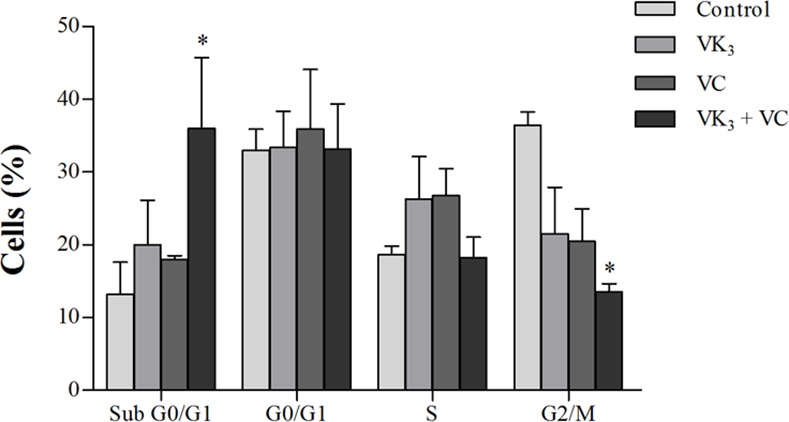
Cell cycle in epimastigote forms of *Trypanosoma cruzi* that were treated with 1.90 μM vitamin K_3_ (VK_3_) and vitamin C (VC), alone and combined, for 24 h, evaluated by flow cytometry. The results are expressed as the mean percentage of cells in each stage of the cell cycle (± SE) of at least three independent experiments. * Indicate significant differences compared with the control group (untreated cells; *p* ≤ 0.05).

We evaluated the effects of VK_3_ and VC, alone and in combination, on the cell cycle of epimastigote forms of *T*. *cruzi* using PI. The parasites that were treated with the VK_3_ + VC combination exhibited a significant percentage (36.0%) of cells in the sub-G0/G1 phase (nuclear DNA and/or mitochondrial DNA fragmentation) compared with 13.2% in the control group and a significant reduction of the percentage (13.5%) of cells in the G2/M phase (DNA duplication) compared with 36.4% in the control group (Fig 9).

### Vitamin K_3_ + vitamin C combination induces the formation of autophagic vacuoles

**Fig 10:**
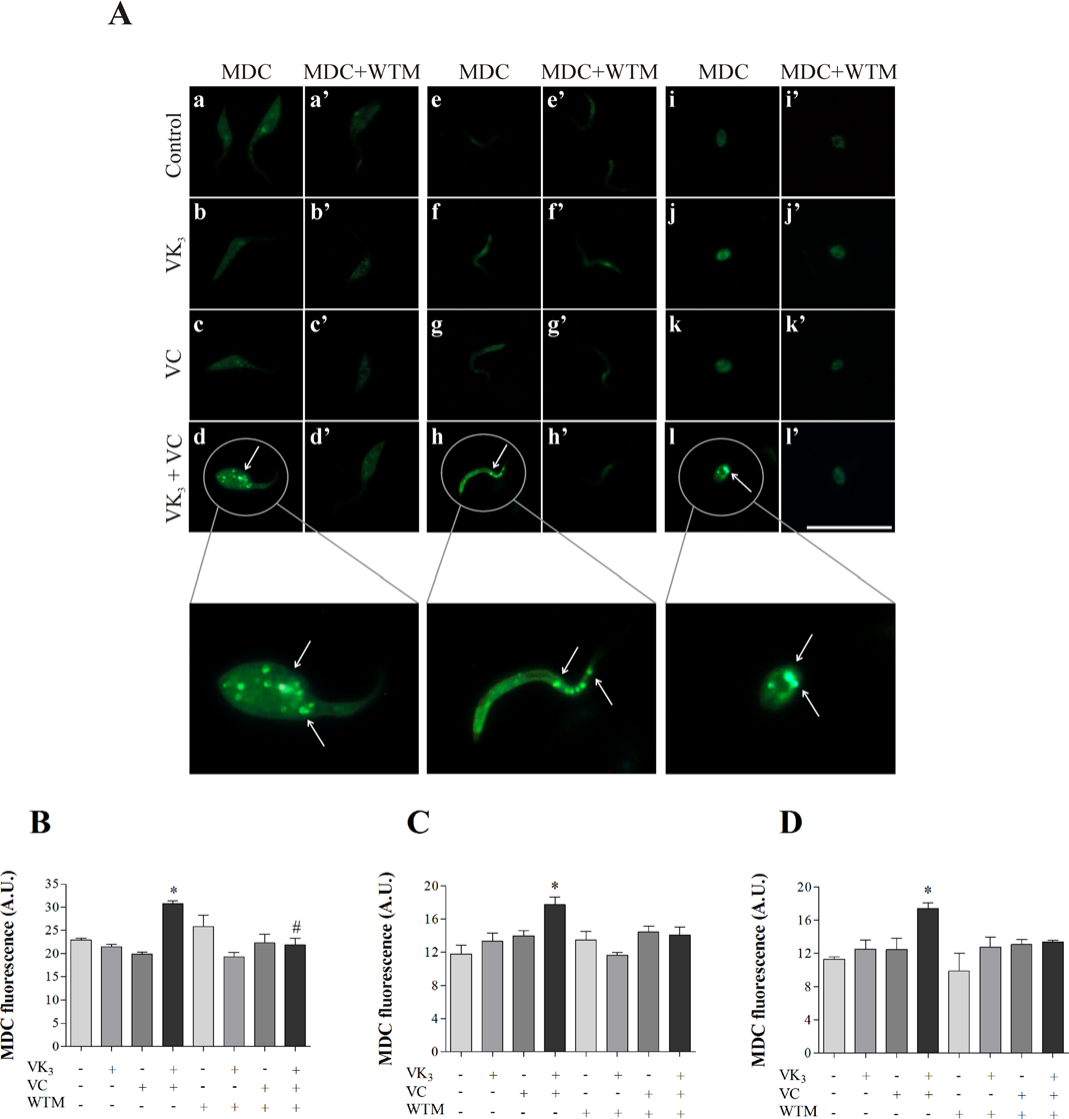
Autophagic vacuoles in parasitic forms of *Trypanosoma cruzi* that were treated with vitamin K_3_ and vitamin C, alone and combined, for 24 h using MDC labeling. (A) MDC fluorescence microscopy images: (a, a’) Untreated epimastigote forms. (b, b’) Epimastigote forms that were treated with 1.90 μM VK_3_. (c, c’) Epimastigote forms that were treated with 0.61 mM VC. (d, d’) Epimastigote forms that were treated with 1.90 μM VK_3_ + 0.61 mM VC. (e, e’) Untreated trypomastigote forms. (f, f’) Trypomastigote forms that were treated with 0.35 μM VK_3_. (g, g’) Trypomastigote forms that were treated with 0.20 mM VC. (h, h’) Trypomastigote forms that were treated with 0.35 μM VK_3_ + 0.20 mM VC. (i, i’) Untreated amastigote forms. (j, j’) Amastigote forms that were treated with 0.30 μM VK_3_. (k, k’) Amastigote forms that were treated with 0.18 mM VC. (l, l’) Amastigote forms that were treated with 0.30 μM VK_3_ + 0.18 mM VC. Arrows, stained autophagic vacuoles; MDC, monodansylcadaverine; MDC + WTM: monodansylcadaverine + wortmannin. Scale bars: 20 μm. (B-D) MDC fluorescence obtained by ImageJ: (B) Epimastigote forms. (C) Trypomastigote forms. (D) Amastigote forms. * Indicate significant differences compared with the control group (untreated cells; *p* ≤ 0.05) and # indicate significant difference compared with the vitamins in combination without WTM (*p* ≤ 0.05).

We evaluated whether autophagy is the cell death process that is induced by VK_3_ + VC in *T*. *cruzi* using MDC labeling. Fig 10 shows that the VK_3_ + VC combination induced the presence of MDC-labeled structure accumulation in the three parasitic forms (Fig 10D, 10H, 10L and 10B, 10C, 10D). More autophagic vacuoles were induced by the vitamin combination compared with either vitamin alone (Fig 10B and 10C, 10F and 10G, 10J and 10K) and the control group (Fig 10A, 10E and 10I). This effect was partially prevented in the parasites that were pretreated with WTM (Fig 10D’, 10H’, 10L’ and 10B, 10C, 10D). The increase in the formation of autophagic vacuoles was significant in epimastigotes (34.0% Fig 10B), trypomastigotes (50.2% Fig 10C), and amastigotes (54.3% Fig 10D).

## Discussion

Several studies have demonstrated the potential of VK_3_ + VC combinations against several cancer cells [18–25], which induce pro-oxidative imbalance through the generation of reactive species [42]. It is well described that the non-enzymatic reduction of VK_3_ by ascorbate leads to VK_3_ semiquinone and ascorbyl free radicals. Then, VK_3_ semiquinone is reoxidized to its quinone form by molecular oxygen, which is, consequently, reduced to O_2_^•−^. From this ROS, others as H_2_O_2_ and HO^●^ can be generated [26]. Thus, Apatone^®^ (one VK_3_ + VC combination) was developed as a new therapeutic strategy for cancer treatment [42, 43]. Previous studies have also reported that the VK_3_ + VC combination has low systemic toxicity [20, 44] and may have different biochemical targets, depending on the cellular type. Combination therapy has been shown to be an important approach for many diseases other than cancer, including such infectious diseases as American trypanosomiasis, which constitutes a serious public health problem [45, 46]. One study demonstrated the effectiveness of a VC + vitamin B_12_ combination against epimastigote forms of *T*. *cruzi*, in which the presence of VC reduced the IC_50_ of vitamin B_12_ [47]. Other studies highlighted the role of VC in combination with vitamin E in attenuating the deleterious effects of chronic inflammatory processes in Chagas’ disease [48] and the activity of VK_3_ against bloodstream trypomastigotes [49].

Considering the different antioxidant capacity of *T*. *cruzi* parasites compared with mammals [30, 31], the present study investigated whether VK_3_ + VC has activity against the epimastigote, trypomastigote, and amastigote forms of *T*. *cruzi*. The most important finding of the present study was the synergistic effects of this vitamin combination against *T*. *cruzi*. We also showed the likely mechanism by which these vitamins exert their trypanocidal effects.

By SEM and TEM we showed that the VK_3_ + VC combination induced several alterations in *T*. *cruzi* parasites, but the most characteristic lesion was swelling in the mitochondrion-kinetoplast region. Previous studies reported the potent effects of naphthoquinones and its derivatives on mitochondrial function [50, 51]. Importantly, morphological or physiological changes in mitochondria can induce a series of harmful events that drive cell death [52, 53]. We also found that the VK_3_ + VC combination induced oxidative imbalance, demonstrated by two findings: increase in reactive species production and a decrease in reduced thiol levels. This vitamin combination is well known to induce a redox cycling process [26, 27]. Additionally, the marked increase in ROS might overload the antioxidant defense system of *T*. *cruzi*, followed by the depletion of key antioxidant enzymes, such as TR, reflected herein by the decrease in reduced thiol levels. Thus, we believe that the increase in ROS formation via redox cycling might be the main effector mechanism of VK_3_ + VC that interferes with maintaining redox homeostasis and mediates *T*. *cruzi* death. *T*. *cruzi* also has the ability to synthesize and capture VC [54], and VK_3_ is able to cross the membrane of the parasite. Based on this, ROS production could occur not only outside the parasite but also inside the parasite, which could explain the high efficacy of this vitamin combination. Other notable characteristics of *T*. *cruzi* are its single mitochondrion [55] and different antioxidant system compared with mammalian counterparts [30, 31, 56], which can be overwhelmed by increases in ROS/NO formation [57]. Studies suggest that naphthoquinones, such as menadione, have been shown to be inhibitors of the *T*. *cruzi* thiol-redox system [58]. Interestingly, alterations in oxidative metabolism in the parasite were more marked in trypomastigotes (i.e., the infective flagellate form), with higher levels of trypanothione, the main dithiol in antioxidant metabolism, compared with epimastigotes (which do not have polyamine supplements) and amastigotes [59]. However, epimastigotes possess a higher content of total thiols, followed by trypomastigotes and amastigotes [59, 60].

Additionally, VK_3_ + VC induced the lipid peroxidation and arrested the cell cycle of *T*. *cruzi*. Alterations in the composition of certain lipids may impair the cell cycle of parasites [61]. The cell cycle process is a key mechanism that regulates cytokinesis to maintain genetic integrity. Cell cycle arrest has been previously shown to be caused by VK_3_ [62] and VK_3_ + VC [22]. The VK_3_ + VC combination also induced the formation of autophagic vacuoles. An increase in reactive species might be a critical event that triggers lipid oxidation, and this alteration may activate cell autophagy machinery [63]. All of the effects of the VK_3_ + VC combination against *T*. *cruzi* described herein appear to be part of a cascade of events that is triggered by redox imbalance, leading to the loss of homeostasis and culminating in parasite death.

In summary, the present study demonstrated the synergic trypanocidal effect of VK_3_ + VC on the three forms of *T*. *cruzi*. These effects were marked by morphological, ultrastructural, and functional changes in this parasite. The data clearly indicate the potential therapeutic utility of this combination for the treatment of Chagas’ disease and open the way for further studies of Apatone^®^ and other naphthoquinones combined with VC.

## Supporting Information

S1 Table Activity of vitamins K_3_ and C, alone and in combination, on *Trypanosoma cruzi*. (PDF)
