# Supplementary material for: The Combination of Vitamin K3 and Vitamin C Has Synergic Activity against Forms of Trypanosoma cruzi through a Redox Imbalance Process
Source: PLoS One. 2015 Dec 7;10(12):e0144033. doi: 10.1371/journal.pone.0144033 (PMC4671608; doi:10.1371/journal.pone.0144033)
Supplement: S1 Table — (PDF) [file pone.0144033.s001.pdf]

S1 Table - Activity of vitamins K_3 (VK_3; µM) and C (VC; mM), alone and in combination, on *Trypanosoma cruzi*.

**Forms**	**VK_3**	**VC**	**Synergistic Combination (VK_3 + VC)**
**Epimastigote (IC_{50})**	3.50	2.0	1.90 + 0.61
**Trypomastigote (EC_{50})**	2.30	0.44	0.35 + 0.20
**Intracellular Amastigote (IC_{50})**	2.0	0.64	0.30 + 0.18

The IC_{50} value represents the concentration that inhibits cell growth in 50%, and the EC_{50} value represents the concentration that lyses 50% of the parasites.
